# Crystal structure and Hirshfeld surface analysis of *N*,*N*′-bis­(2-nitro­phen­yl)glutaramide

**DOI:** 10.1107/S2056989018013075

**Published:** 2018-09-21

**Authors:** Akshatha R. Salian, Sabine Foro, S. Madan Kumar, B. Thimme Gowda

**Affiliations:** a Department of Chemistry, Mangalore University, Mangalagangotri 574 199, India; b Institute of Materials Science, Darmstadt University of Technology, Alarich-Weiss-Str. 2, D-64287, Darmstadt, Germany; c PURSE Lab, Mangalore University, Mangalagangothri 574 199, India; d Karnataka State Rural Development and Panchayat Raj University, Gadag 582 101, India

**Keywords:** crystal structure, bis-amides, inter­molecular hydrogen bonds, Hirshfeld surface analysis

## Abstract

The asymmetric unit of the title compound, C_17_H_16_N_4_O_6_, contains two independent mol­ecules (*A* and *B*). The two benzene rings are twisted by an angle of 79.14 (7)° in mol­ecule *A*, whereas, in mol­ecule *B*, they are inclined by 19.02 (14)°. The conformations of the mol­ecules are stabilized by intra­molecular N—H⋯O hydrogen bonds between the amide nitro­gen atom and the O atom of the *ortho*-nitro substituent on the phenyl ring, enclosing an *S*(6) ring motif. In the amide and aliphatic segments, all the N—H, C=O and C—H bonds are *anti* to each other. In the crystal, the *A* and *B* mol­ecules are linked by inter­molecular amide-to-amide N—H⋯O hydrogen bonds, resulting in chains running along the *b-*axis direction. The inter­molecular inter­actions were analysed using Hirshfeld surface analysis. The two-dimensional fingerprint plots of the inter­molecular contacts indicate that the major contributions are from H⋯H and O⋯H inter­actions.

## Abstract 2

The title bis-amide derivative was obtained by the reaction between glutaric acid chloride and 2-nitro­aniline. The two benzene rings are twisted by angles of 79.14 (7) and 19.02 (14)° in the two independent mol­ecules. In the crystal, amide–amide inter­actions link the mol­ecules into chains running along *b*-axis direction.

## Chemical context   

Alkanedi­amide derivatives are known to possess a variety of biological activities. There has been a study on the influence of the length of the connecting chain on the anti­malarial activity of bis­quinolines (Raynes *et al.*, 1995 ▸) and OER (oxygen evolution rate) inhibiting activity in spinach in a series of *N,N′*-bis­(3,4-di­chloro­phen­yl)alkanedi­amides (Kubicova *et al.*, 2000*a* ▸,*b* ▸). The crystal structures of a homologous series of bis­(pyridine­carboxamido)­alkanes have been studied to analyse their supra­molecular structures (Sarkar & Biradha, 2006 ▸). As a part of a study on substituent effects on the structures of bis-amides, the crystal structure of *N,N′*-bis­(2-nitro­phen­yl)glutaramide has been determined and is described in the present work.
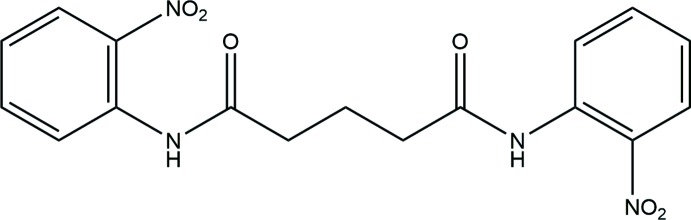


## Structural commentary   

**Figure 1:**
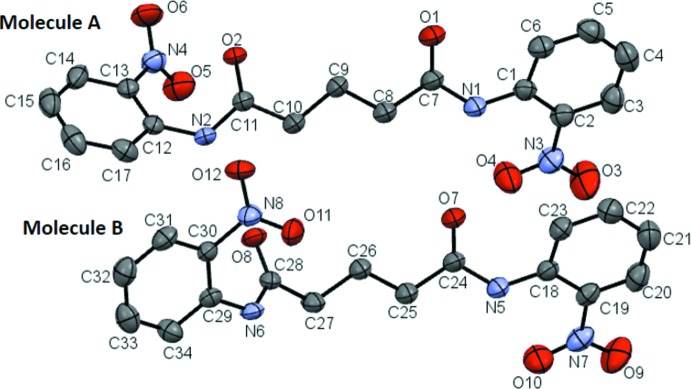
Mol­ecular structure of (I), showing the atom-labelling scheme. Displacement ellipsoids are drawn at the 50% probability level and hydrogen atoms are omitted for clarity.

The asymmetric unit of the title compound (I) contains two independent mol­ecules (designated as *A* and *B* in Fig. 1 ▸), and four mol­ecules in the unit cell. In both the mol­ecules present in the asymmetric unit, all the N—H, C=O and C—H bonds of the amide and aliphatic segments are *anti* to each other. The conformation of the nearest C=O group is *anti* to the *ortho*-nitro group in the aniline ring in one half of each mol­ecule, as indicated by the torsion angles of −159.5 (3) and −161.9 (3)° for C2—C1—N1—C7 and C19—C18—N5—C24, respectively. In the other half, they are *syn* to the *ortho*-substituent as shown by the torsion angles of 48.6 (4) and −50.6 (4)° for C13—C12—N2—C11 and C30—C29—N6—C28, respectively. The O1—C7, O2—C11, O7—C24 and O8—C28 bond lengths are 1.213 (3), 1.224 (3), 1.218 (3) and 1.218 (3) Å, respectively, which indicate that the mol­ecules exist in their keto forms in the solid state. In mol­ecule *A*, the bis-amide group forms dihedral angles of 24.79 (12) and 55.04 (7)° with the phenyl rings C1–C6 and C12–C17, respectively. In mol­ecule *B*, the plane of the amide group forms dihedral angles of 34.24 (13) and 24.27 (12)° with the C18–C23 and C29–C34 phenyl rings, respectively, while the two benzene rings form a dihedral angle of 79.14 (7) and 19.02 (14)° in mol­ecules *A* and *B*, respectively. The planes of mol­ecules *A* and *B* are almost coplanar with each other, as is evident from the dihedral angle of only 3.15 (17)° between phenyl rings C1–C6 and C18–C23.

The O atoms of the *ortho*-substituted nitro groups attached to the C1/C6 and C18/C23 phenyl rings form short intra­molecular contacts, each of 2.01 (3) Å, with the nearest amide N atom, forming an N—H⋯O contact resulting in an *S*(6) hydrogen bonding motif.

## Supra­molecular features   

**Figure 2:**
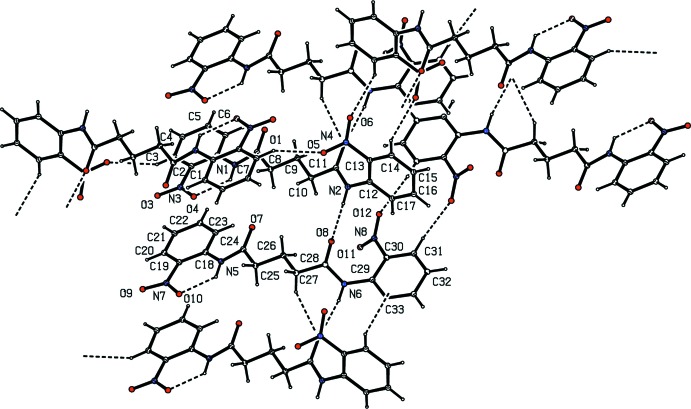
Hydrogen-bonding pattern in (I) with hydrogen bonds shown as dashed lines.

**Figure 3:**
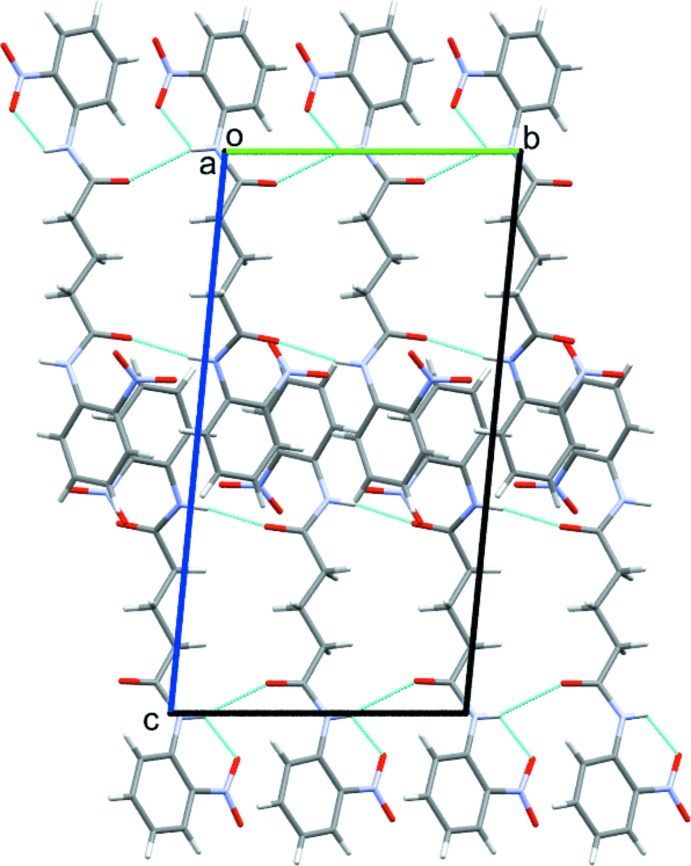
Mol­ecular packing of (I) with hydrogen bonds shown as dashed lines.

In the crystal, the mol­ecules are linked by N—H⋯O and C—H⋯O hydrogen bonds (Table 1 ▸ and Fig. 2 ▸). An inter­molecular amide-to-amide N—H⋯O hydrogen bond between two bis-amide groups results in mol­ecular chains running along the *b-*axis direction. The oxygen atom of the amide C=O group in mol­ecule *B* forms a bifurcated hydrogen bond with the N—H group of the amide unit and the C—H group of the aliphatic chain of an adjacent mol­ecule. The C3—H3 unit of the C1–C6 ring of mol­ecule *A* forms a short inter­molecular contact with the oxygen atom O5 belonging to the nitro group of the C12–C17 phenyl ring of another *A* mol­ecule at position −*x*, 1 − *y*, −*z*. C—H groups of the C12–C17 and C29–C34 phenyl rings form hydrogen bonds with the O atoms of the nitro groups of the C12/C17 and C29/C34 phenyl rings at −*x*, −*y* + 2, −*z* + 1 and −*x* + 1, −*y* + 1, −*z* + 1, respectively. A packing diagram of the title compound is shown in Fig. 3 ▸.

## Hirshfeld Surface analysis   

**Figure 4:**
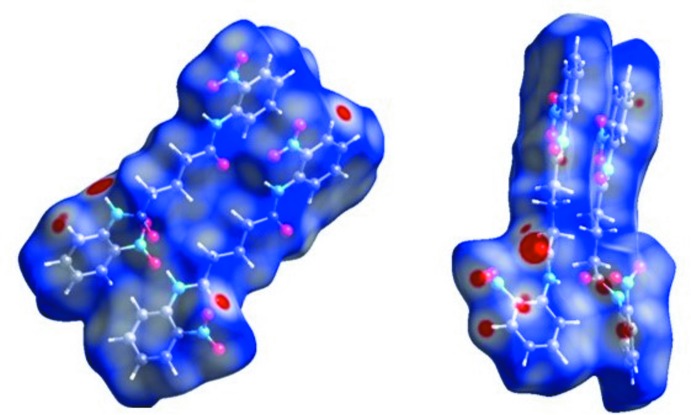
View of the Hirshfeld surface mapped over *d*_norm_ for the two independent mol­ecules (*A* and *B*). The colour scale is between −0.21 au (red) to 1.2 au (blue).

**Figure 5:**
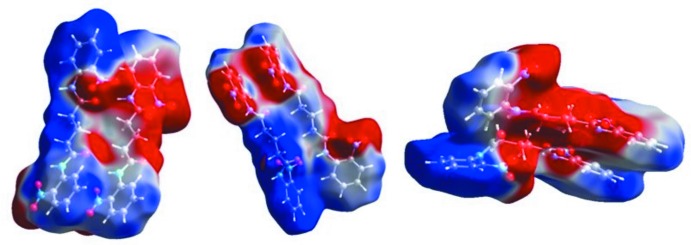
View of the Hirshfeld surface mapped over the electrostatic potential for the two mol­ecules (*A* and *B*).

The inter­molecular contacts in the crystal structure were investigated using Hirshfeld surface analysis and two-dimensional fingerprint plots, generated using *CrystalExplorer* (Figs. 4 ▸, 5 ▸ and 6 ▸). The red-coloured areas of the Hirshfeld surface indicate inter­molecular inter­actions (McKinnon *et al.*, 2004 ▸; Spackman & McKinnon, 2002 ▸; Spackman & Jayatilaka, 2009 ▸; Madan *et al.*, 2013 ▸). Dark-red areas on the *d*_norm_ surface arise as a result of short inter­atomic contacts, *i.e*. strong hydrogen bonds, while the other inter­molecular inter­actions appear as light-red spots (Fig. 4 ▸). In the surface mapped over the electrostatic potential (Fig. 5 ▸), blue and red regions around the atoms correspond to the positive and negative electrostatic potentials of the N—H⋯O and C—H⋯O hydrogen-bond donors and acceptors, respectively.

**Figure 6:**
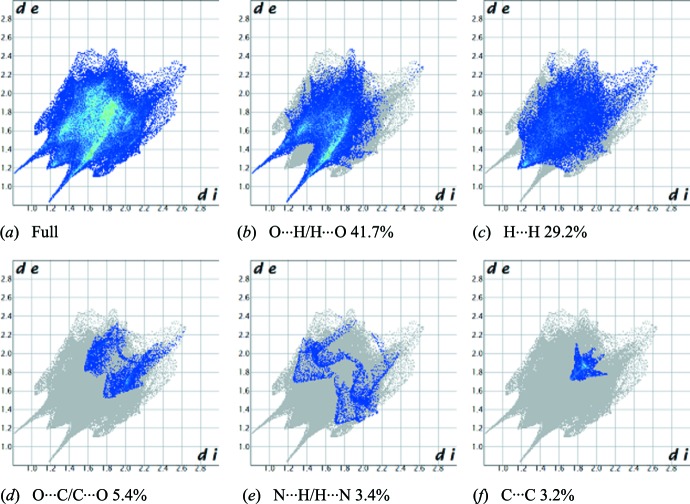
Two-dimensional fingerprint plots for the title compound showing the contributions of different types of inter­actions

In the two-dimensional fingerprint plot (Fig. 6 ▸), *d*_i_ is the closest inter­nal distance from a given point on the Hirshfeld surface to the nearest atom and *d*_e_ is the closest external contact. The outline of the full fingerprint is shown in grey. The fingerprint plots are used to plot inter­molecular contacts with respect to *d*_i_ and *d*_e_. Visualization of the Hirshfeld surfaces and fingerprint plots allow the inter­molecular inter­actions to be qu­anti­fied. The fingerprint plot of O⋯H/H⋯O contacts shows two symmetrical narrow pointed wings, which represent the largest contribution to the Hirshfeld surfaces (41.7%), with *d*_e_ + *d*_i_ ∼ 2.4 Å (Fig. 6 ▸ *b*). H⋯H contacts represent the next largest contribution to the Hirshfeld surfaces (29.2%) and show a distinct pattern with a minimum value of *d*_e_ = *d*_i_ ∼ 1.2 Å (Fig. 6 ▸ *c*). O⋯C/C⋯O and N⋯H/H⋯N inter­actions cover only 5.4% (Fig. 6 ▸ *d*) and 3.4% (Fig. 6 ▸ *e*) of the surface, respectively. Two triangles featuring the C⋯C contacts contribute 3.2% to the Hirshfeld surfaces, with a minimum (*d*_e_ + *d*_i_) distance of 3.5 Å (Fig. 6 ▸ *f*).

## Related structures   

The structure of bis-amides, namely, 3-methyl; 2-chloro-propanedi­amides (Gowda *et al.*, 2010*b* ▸,*c* ▸), *N,N′*-bis­(phen­yl)suberamide (Gowda *et al.*, 2010*a* ▸), bis-2-methyl; 2-chloro; 4-chloro­succinamide (Saraswathi *et al.*, 2011*a* ▸, 2011*b* ▸, Purandara *et al.*, 2012 ▸) and bis-3-chloro­phenyl­malonamide (Rodrigues *et al.*, 2011 ▸) have been investigated as part of our studies on the substituent effect on the structures and other aspects of the bis-amides. The title compound is similar to these compounds with the difference being the length of the aliphatic chain, substituent type and position in the phenyl ring of the mol­ecule.

## Synthesis and crystallization   

A mixture of glutaric acid (0.2 mol) and thionyl chloride (1.0 mol) was heated for half an hour at 363 K. Then 2-nitro­aniline (0.4 mol) was added dropwise under stirring. The resultant mixture was stirred for 3 h and left standing for 12 h for the completion of the reaction. The product was added to crushed ice. The white precipitate obtained was washed thoroughly with water and then with saturated sodium bicarbonate solution and again with water. It was washed first with 2 *N* HCl, then with water, collected by filtration, dried and recrystallized from dimethyl formamide (melting point: 503–504 K). The purity of the compound was checked by TLC and it was characterized by IR spectroscopy. The characteristic absorptions were observed at 3334.9, 1693.5 and 1330.9 cm^−1^ for N—H, C=O and C—N, respectively. ^1^H NMR (400 MHz, DMSO, δ in p.p.m): 1.93 to 2.00 (*q*, 1H, alk­yl–H), 2.48 (*t*, 2H, alk­yl–H, *J* = 7.4 Hz), 7.95 (*dd*, 1H, Ar–H, *J* = 8.2, 1.4 Hz) , 7.28–7.33 (*m*, 1H, Ar–H), 7.63–7.70 (*m*, 1H, Ar–H), 7.82 (*dd*, 1H, Ar–H, *J* = 8.2, 1.2 Hz) , 10.24 (*s*, 1H, –NH–). ^13^C NMR (100 MHz, DMSO, δ in p.p.m): 20.40, 35.19, 124.38, 124.65, 124.68, 131.71, 133.75, 141.36 and 170.82. Rod-shaped yellow single crystals of the title compound were obtained by slow evaporation of a DMF solution at room temperature.

## Refinement   

Crystal data, data collection and structure refinement details are summarized in Table 2 ▸. C-bound H atoms were positioned with idealized geometry [C—H = 0.93 Å or 0.97 Å (methyl­ene)] and refined using a riding model with *U*_iso_(H) = 1.2*U*_eq_(C). The H atoms of the NH groups were located in a difference map and later restrained to a distance of N—H = 0.86 (2) Å. They were refined with *U*_iso_(H) = 1.2 *U*_eq_(N).

## Supplementary Material

Crystal structure: contains datablock(s) I, global. DOI: 10.1107/S2056989018013075/zl2738sup1.cif

Structure factors: contains datablock(s) I. DOI: 10.1107/S2056989018013075/zl2738Isup2.hkl

Supporting information file. DOI: 10.1107/S2056989018013075/zl2738Isup3.cml

CCDC reference: 1578746

Additional supporting information:  crystallographic information; 3D view; checkCIF report

## Figures and Tables

**Table 1:** Hydrogen-bond geometry (Å, °)

*D*—H⋯*A*	*D*—H	H⋯*A*	*D*⋯*A*	*D*—H⋯*A*
N1—H1*N*⋯O4	0.86 (2)	2.01 (3)	2.639 (3)	130 (3)
N2—H2*N*⋯O8	0.86 (2)	2.17 (2)	3.002 (3)	162 (3)
C3—H3⋯O5^i^	0.93	2.49	3.337 (4)	151
C14—H14⋯O6^ii^	0.93	2.50	3.267 (3)	140
N5—H5*N*⋯O10	0.86 (2)	2.01 (3)	2.651 (3)	130 (3)
N6—H6*N*⋯O2^iii^	0.86 (2)	2.11 (2)	2.959 (3)	171 (3)
C27—H27*B*⋯O2^iii^	0.97	2.55	3.396 (3)	146
C31—H31⋯O12^iv^	0.93	2.49	3.238 (3)	138

Symmetry codes: (i) 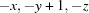; (ii) 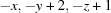; (iii) 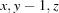; (iv) 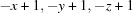.

**Table 2:** Experimental details

Crystal data	
Chemical formula	C_17_H_16_N_4_O_6_
*M*_r_	372.34
Crystal system, space group	Triclinic, *P* 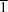
Temperature (K)	293
*a*, *b*, *c* (Å)	9.625 (1), 9.673 (1), 18.500 (2)
α, β, γ (°)	95.37 (1), 93.38 (1), 92.77 (1)
*V* (Å^3^)	1709.3 (3)
*Z*	4
Radiation type	Mo *K*α
μ (mm^−1^)	0.11
Crystal size (mm)	0.48 × 0.26 × 0.06

Data collection	
Diffractometer	Oxford Diffraction Xcalibur with Sapphire CCD
Absorption correction	Multi-scan (*CrysAlis RED*; Oxford Diffraction, 2009 ▸)
*T*_min_, *T*_max_	0.948, 0.993
No. of measured, independent and observed [*I* > 2σ(*I*)] reflections	10696, 6248, 3877
*R*_int_	0.030
(sin θ/λ)_max_ (Å^−1^)	0.602

Refinement	
*R*[*F* ^2^ > 2σ(*F* ^2^)], *wR*(*F* ^2^), *S*	0.058, 0.158, 1.05
No. of reflections	6248
No. of parameters	499
No. of restraints	4
H-atom treatment	H atoms treated by a mixture of independent and constrained refinement
Δρ_max_, Δρ_min_ (e Å^−3^)	0.27, −0.22

Computer programs: *CrysAlis CCD* and *CrysAlis RED* (Oxford Diffraction, 2009 ▸), *SHELXS2013/1* (Sheldrick, 2008 ▸), *SHELXL2014/6* (Sheldrick, 2015 ▸) and *PLATON* (Spek, 2003 ▸).

## supplementary crystallographic information

### Crystal data

**Table:**

C_17_H_16_N_4_O_6_	*Z* = 4
*M**_r_* = 372.34	*F*(000) = 776
Triclinic, *P*1	*D*_x_ = 1.447 Mg m^−^^3^
*a* = 9.625 (1) Å	Mo *K*α radiation, λ = 0.71073 Å
*b* = 9.673 (1) Å	Cell parameters from 2127 reflections
*c* = 18.500 (2) Å	θ = 2.9–27.7°
α = 95.37 (1)°	µ = 0.11 mm^−^^1^
β = 93.38 (1)°	*T* = 293 K
γ = 92.77 (1)°	Rod, yellow
*V* = 1709.3 (3) Å^3^	0.48 × 0.26 × 0.06 mm

### Data collection

**Table:**

Oxford Diffraction Xcalibur with Sapphire CCD diffractometer	3877 reflections with *I* > 2σ(*I*)
Radiation source: Enhance (Mo) X-ray Source	*R*_int_ = 0.030
Rotation method data acquisition using ω scans.	θ_max_ = 25.4°, θ_min_ = 2.9°
Absorption correction: multi-scan (CrysAlis RED; Oxford Diffraction, 2009)	*h* = −11→11
*T*_min_ = 0.948, *T*_max_ = 0.993	*k* = −11→6
10696 measured reflections	*l* = −20→22
6248 independent reflections	

### Refinement

**Table:**

Refinement on *F*^2^	Primary atom site location: structure-invariant direct methods
Least-squares matrix: full	Secondary atom site location: difference Fourier map
*R*[*F*^2^ > 2σ(*F*^2^)] = 0.058	Hydrogen site location: mixed
*wR*(*F*^2^) = 0.158	H atoms treated by a mixture of independent and constrained refinement
*S* = 1.05	*w* = 1/[σ^2^(*F*_o_^2^) + (0.0698*P*)^2^ + 0.4948*P*] where *P* = (*F*_o_^2^ + 2*F*_c_^2^)/3
6248 reflections	(Δ/σ)_max_ < 0.001
499 parameters	Δρ_max_ = 0.27 e Å^−^^3^
4 restraints	Δρ_min_ = −0.21 e Å^−^^3^

### Special details

**Table:**

Geometry. All esds (except the esd in the dihedral angle between two l.s. planes) are estimated using the full covariance matrix. The cell esds are taken into account individually in the estimation of esds in distances, angles and torsion angles; correlations between esds in cell parameters are only used when they are defined by crystal symmetry. An approximate (isotropic) treatment of cell esds is used for estimating esds involving l.s. planes.

### Fractional atomic coordinates and isotropic or equivalent isotropic displacement parameters (Å^2^)

**Table:**

	*x*	*y*	*z*	*U*_iso_*/*U*_eq_	
O1	0.3147 (3)	0.67820 (19)	0.05292 (11)	0.0655 (7)	
O2	0.2237 (2)	0.73540 (16)	0.33207 (10)	0.0451 (5)	
O3	0.0896 (3)	0.2146 (3)	−0.18879 (14)	0.1000 (10)	
O4	0.0869 (3)	0.2646 (3)	−0.07551 (13)	0.0799 (8)	
O5	−0.0882 (2)	0.6967 (2)	0.35971 (12)	0.0655 (6)	
O6	−0.0156 (2)	0.9019 (2)	0.40685 (13)	0.0644 (6)	
N1	0.2543 (3)	0.4663 (2)	−0.00794 (13)	0.0491 (6)	
H1N	0.206 (3)	0.391 (2)	−0.0022 (16)	0.059*	
N2	0.1565 (3)	0.5376 (2)	0.37960 (12)	0.0408 (6)	
H2N	0.154 (3)	0.4480 (18)	0.3730 (15)	0.049*	
N3	0.1320 (3)	0.2859 (3)	−0.13395 (15)	0.0515 (6)	
N4	−0.0203 (2)	0.7745 (2)	0.40554 (13)	0.0452 (6)	
C1	0.2958 (3)	0.4828 (3)	−0.07765 (14)	0.0393 (6)	
C2	0.2399 (3)	0.3959 (3)	−0.13878 (15)	0.0408 (7)	
C3	0.2840 (3)	0.4113 (3)	−0.20821 (16)	0.0543 (8)	
H3	0.2461	0.3520	−0.2477	0.065*	
C4	0.3825 (4)	0.5128 (4)	−0.21869 (18)	0.0607 (9)	
H4	0.4111	0.5237	−0.2651	0.073*	
C5	0.4389 (3)	0.5990 (3)	−0.15941 (18)	0.0551 (8)	
H5	0.5061	0.6682	−0.1663	0.066*	
C6	0.3978 (3)	0.5847 (3)	−0.09057 (17)	0.0485 (7)	
H6	0.4384	0.6437	−0.0517	0.058*	
C7	0.2653 (3)	0.5604 (3)	0.05253 (15)	0.0424 (7)	
C8	0.2078 (3)	0.5029 (3)	0.11788 (15)	0.0480 (7)	
H8A	0.2476	0.4141	0.1238	0.058*	
H8B	0.1079	0.4858	0.1088	0.058*	
C9	0.2354 (3)	0.5960 (3)	0.18866 (14)	0.0441 (7)	
H9A	0.3341	0.6228	0.1956	0.053*	
H9B	0.1850	0.6800	0.1859	0.053*	
C10	0.1911 (4)	0.5236 (3)	0.25192 (15)	0.0541 (8)	
H10A	0.2503	0.4462	0.2572	0.065*	
H10B	0.0966	0.4849	0.2406	0.065*	
C11	0.1953 (3)	0.6099 (2)	0.32375 (14)	0.0361 (6)	
C12	0.1367 (3)	0.5978 (2)	0.45035 (14)	0.0336 (6)	
C13	0.0578 (3)	0.7132 (3)	0.46469 (14)	0.0360 (6)	
C14	0.0414 (3)	0.7718 (3)	0.53445 (16)	0.0482 (8)	
H14	−0.0110	0.8496	0.5418	0.058*	
C15	0.1025 (3)	0.7150 (3)	0.59281 (16)	0.0548 (8)	
H15	0.0925	0.7541	0.6400	0.066*	
C16	0.1794 (3)	0.5988 (3)	0.58054 (16)	0.0546 (8)	
H16	0.2198	0.5586	0.6198	0.065*	
C17	0.1970 (3)	0.5415 (3)	0.51009 (16)	0.0451 (7)	
H17	0.2501	0.4642	0.5029	0.054*	
O7	0.3045 (3)	0.17649 (19)	0.05705 (11)	0.0663 (7)	
O8	0.2036 (2)	0.23692 (17)	0.33766 (10)	0.0448 (5)	
O9	0.0953 (3)	−0.2729 (3)	−0.19779 (13)	0.0802 (8)	
O10	0.0912 (3)	−0.2415 (2)	−0.08189 (13)	0.0700 (7)	
O11	0.5169 (2)	0.2177 (2)	0.34074 (12)	0.0629 (6)	
O12	0.4749 (2)	0.41508 (19)	0.39786 (11)	0.0568 (6)	
N5	0.2408 (3)	−0.0328 (2)	−0.00706 (13)	0.0455 (6)	
H5N	0.197 (3)	−0.112 (2)	−0.0042 (16)	0.055*	
N6	0.2854 (2)	0.0364 (2)	0.37197 (12)	0.0376 (5)	
H6N	0.272 (3)	−0.0524 (18)	0.3653 (14)	0.045*	
N7	0.1378 (3)	−0.2110 (2)	−0.13923 (15)	0.0509 (6)	
N8	0.4744 (2)	0.2875 (2)	0.39265 (13)	0.0432 (6)	
C18	0.2939 (3)	−0.0158 (3)	−0.07437 (14)	0.0386 (6)	
C19	0.2469 (3)	−0.1001 (3)	−0.13854 (15)	0.0407 (7)	
C20	0.3026 (3)	−0.0821 (3)	−0.20481 (16)	0.0559 (8)	
H20	0.2700	−0.1393	−0.2461	0.067*	
C21	0.4051 (4)	0.0189 (3)	−0.20990 (17)	0.0592 (9)	
H21	0.4422	0.0306	−0.2544	0.071*	
C22	0.4527 (3)	0.1035 (3)	−0.14813 (17)	0.0530 (8)	
H22	0.5213	0.1733	−0.1514	0.064*	
C23	0.3996 (3)	0.0855 (3)	−0.08174 (15)	0.0463 (7)	
H23	0.4351	0.1422	−0.0408	0.056*	
C24	0.2492 (3)	0.0601 (3)	0.05446 (15)	0.0422 (7)	
C25	0.1819 (3)	0.0042 (3)	0.11826 (14)	0.0429 (7)	
H25A	0.2108	−0.0894	0.1229	0.052*	
H25B	0.0815	−0.0008	0.1089	0.052*	
C26	0.2196 (3)	0.0934 (3)	0.18947 (14)	0.0410 (7)	
H26A	0.1857	0.1855	0.1859	0.049*	
H26B	0.3202	0.1029	0.1975	0.049*	
C27	0.1582 (3)	0.0320 (3)	0.25438 (14)	0.0434 (7)	
H27A	0.0575	0.0352	0.2503	0.052*	
H27B	0.1811	−0.0645	0.2545	0.052*	
C28	0.2149 (3)	0.1123 (2)	0.32459 (14)	0.0340 (6)	
C29	0.3407 (3)	0.0933 (2)	0.44173 (14)	0.0334 (6)	
C30	0.4243 (3)	0.2158 (2)	0.45335 (14)	0.0348 (6)	
C31	0.4704 (3)	0.2724 (3)	0.52307 (16)	0.0463 (7)	
H31	0.5248	0.3553	0.5295	0.056*	
C32	0.4354 (3)	0.2058 (3)	0.58253 (16)	0.0539 (8)	
H32	0.4655	0.2433	0.6293	0.065*	
C33	0.3553 (3)	0.0829 (3)	0.57198 (16)	0.0538 (8)	
H33	0.3325	0.0367	0.6119	0.065*	
C34	0.3083 (3)	0.0273 (3)	0.50262 (15)	0.0448 (7)	
H34	0.2542	−0.0557	0.4967	0.054*	

### Atomic displacement parameters (Å^2^)

**Table:**

	*U*^11^	*U*^22^	*U*^33^	*U*^12^	*U*^13^	*U*^23^
O1	0.111 (2)	0.0311 (11)	0.0515 (13)	−0.0148 (12)	0.0110 (12)	−0.0030 (9)
O2	0.0691 (13)	0.0216 (9)	0.0441 (11)	−0.0060 (9)	0.0122 (10)	−0.0012 (8)
O3	0.111 (2)	0.118 (2)	0.0579 (17)	−0.0585 (19)	−0.0034 (15)	−0.0241 (15)
O4	0.0968 (19)	0.0837 (17)	0.0527 (16)	−0.0467 (15)	0.0060 (14)	−0.0017 (13)
O5	0.0732 (16)	0.0596 (14)	0.0594 (15)	0.0004 (12)	−0.0224 (12)	0.0010 (11)
O6	0.0776 (16)	0.0361 (12)	0.0822 (17)	0.0150 (11)	0.0116 (13)	0.0100 (11)
N1	0.0760 (19)	0.0321 (12)	0.0369 (14)	−0.0132 (12)	0.0053 (12)	−0.0010 (11)
N2	0.0626 (16)	0.0208 (10)	0.0395 (14)	0.0009 (11)	0.0136 (11)	0.0003 (10)
N3	0.0525 (16)	0.0524 (15)	0.0454 (16)	−0.0078 (13)	−0.0075 (13)	−0.0049 (13)
N4	0.0467 (15)	0.0373 (14)	0.0520 (16)	0.0063 (11)	0.0070 (12)	0.0020 (12)
C1	0.0477 (17)	0.0326 (14)	0.0374 (16)	0.0024 (12)	−0.0005 (13)	0.0045 (12)
C2	0.0438 (16)	0.0379 (15)	0.0400 (17)	0.0018 (13)	−0.0035 (13)	0.0032 (12)
C3	0.062 (2)	0.064 (2)	0.0360 (18)	0.0035 (17)	−0.0036 (15)	0.0015 (15)
C4	0.066 (2)	0.074 (2)	0.046 (2)	0.0075 (19)	0.0119 (17)	0.0167 (17)
C5	0.0527 (19)	0.0509 (18)	0.065 (2)	0.0003 (15)	0.0138 (16)	0.0166 (16)
C6	0.0539 (18)	0.0393 (16)	0.0509 (19)	−0.0059 (14)	0.0024 (14)	0.0017 (13)
C7	0.0597 (19)	0.0290 (14)	0.0371 (16)	−0.0001 (13)	−0.0012 (13)	0.0008 (12)
C8	0.069 (2)	0.0327 (15)	0.0414 (17)	−0.0047 (14)	0.0072 (15)	−0.0005 (12)
C9	0.066 (2)	0.0299 (14)	0.0355 (16)	−0.0008 (13)	0.0070 (14)	−0.0023 (12)
C10	0.092 (2)	0.0304 (15)	0.0388 (17)	−0.0057 (15)	0.0142 (16)	−0.0030 (12)
C11	0.0445 (16)	0.0273 (14)	0.0367 (15)	0.0010 (12)	0.0086 (12)	0.0016 (11)
C12	0.0343 (14)	0.0313 (13)	0.0349 (15)	−0.0054 (11)	0.0070 (11)	0.0031 (11)
C13	0.0397 (15)	0.0316 (14)	0.0355 (15)	−0.0061 (12)	0.0040 (12)	0.0003 (11)
C14	0.0534 (18)	0.0427 (16)	0.0459 (18)	−0.0076 (14)	0.0140 (15)	−0.0117 (14)
C15	0.059 (2)	0.067 (2)	0.0349 (18)	−0.0189 (17)	0.0070 (15)	−0.0050 (15)
C16	0.0450 (18)	0.077 (2)	0.0412 (19)	−0.0155 (17)	−0.0016 (14)	0.0176 (16)
C17	0.0406 (16)	0.0461 (17)	0.0506 (19)	−0.0011 (13)	0.0056 (14)	0.0147 (14)
O7	0.118 (2)	0.0311 (11)	0.0479 (13)	−0.0172 (12)	0.0230 (13)	−0.0062 (9)
O8	0.0652 (13)	0.0241 (10)	0.0440 (12)	0.0046 (9)	−0.0024 (9)	0.0004 (8)
O9	0.0763 (17)	0.0865 (17)	0.0662 (17)	−0.0178 (14)	−0.0146 (13)	−0.0306 (13)
O10	0.0775 (17)	0.0651 (15)	0.0622 (16)	−0.0260 (13)	0.0001 (13)	−0.0028 (12)
O11	0.0771 (16)	0.0529 (13)	0.0581 (14)	−0.0100 (11)	0.0341 (12)	−0.0102 (11)
O12	0.0718 (15)	0.0289 (11)	0.0686 (15)	−0.0100 (10)	0.0086 (11)	0.0032 (9)
N5	0.0667 (17)	0.0322 (12)	0.0354 (14)	−0.0096 (12)	0.0028 (12)	−0.0016 (10)
N6	0.0516 (14)	0.0201 (10)	0.0393 (13)	−0.0046 (10)	−0.0016 (11)	0.0001 (10)
N7	0.0477 (15)	0.0455 (14)	0.0551 (17)	0.0016 (12)	−0.0094 (13)	−0.0094 (13)
N8	0.0452 (14)	0.0335 (13)	0.0494 (15)	−0.0088 (11)	0.0105 (11)	−0.0035 (11)
C18	0.0478 (17)	0.0310 (14)	0.0366 (16)	0.0050 (12)	0.0008 (13)	0.0000 (11)
C19	0.0445 (16)	0.0370 (15)	0.0385 (16)	0.0058 (13)	−0.0044 (13)	−0.0048 (12)
C20	0.065 (2)	0.061 (2)	0.0389 (19)	0.0070 (17)	−0.0041 (15)	−0.0070 (15)
C21	0.068 (2)	0.067 (2)	0.0425 (19)	0.0055 (18)	0.0116 (16)	0.0030 (16)
C22	0.057 (2)	0.0473 (18)	0.055 (2)	−0.0008 (15)	0.0095 (16)	0.0054 (15)
C23	0.0570 (19)	0.0407 (16)	0.0390 (17)	−0.0041 (14)	0.0023 (14)	−0.0036 (12)
C24	0.0616 (19)	0.0280 (14)	0.0365 (16)	0.0020 (13)	0.0025 (13)	0.0011 (12)
C25	0.0571 (18)	0.0327 (14)	0.0378 (16)	−0.0053 (13)	0.0037 (13)	0.0010 (12)
C26	0.0541 (18)	0.0319 (14)	0.0357 (16)	−0.0045 (13)	0.0028 (13)	0.0004 (11)
C27	0.0599 (19)	0.0324 (14)	0.0361 (16)	−0.0104 (13)	−0.0020 (13)	0.0042 (12)
C28	0.0423 (15)	0.0261 (14)	0.0332 (15)	−0.0056 (11)	0.0053 (12)	0.0031 (11)
C29	0.0340 (14)	0.0278 (13)	0.0381 (15)	0.0042 (11)	0.0017 (11)	0.0006 (11)
C30	0.0396 (15)	0.0287 (13)	0.0357 (15)	−0.0003 (11)	0.0076 (12)	−0.0016 (11)
C31	0.0464 (17)	0.0389 (15)	0.0501 (19)	−0.0007 (13)	−0.0019 (14)	−0.0092 (13)
C32	0.059 (2)	0.062 (2)	0.0387 (18)	0.0054 (17)	−0.0029 (15)	−0.0039 (15)
C33	0.0527 (19)	0.070 (2)	0.0409 (18)	0.0060 (16)	0.0005 (14)	0.0147 (15)
C34	0.0448 (17)	0.0435 (16)	0.0466 (18)	−0.0057 (13)	−0.0011 (14)	0.0127 (13)

### Geometric parameters (Å, º)

**Table:**

O1—C7	1.213 (3)	O7—C24	1.218 (3)
O2—C11	1.224 (3)	O8—C28	1.218 (3)
O3—N3	1.210 (3)	O9—N7	1.225 (3)
O4—N3	1.220 (3)	O10—N7	1.231 (3)
O5—N4	1.217 (3)	O11—N8	1.223 (3)
O6—N4	1.228 (3)	O12—N8	1.229 (3)
N1—C7	1.370 (3)	N5—C24	1.378 (3)
N1—C1	1.393 (3)	N5—C18	1.394 (3)
N1—H1N	0.863 (17)	N5—H5N	0.862 (17)
N2—C11	1.361 (3)	N6—C28	1.366 (3)
N2—C12	1.409 (3)	N6—C29	1.417 (3)
N2—H2N	0.863 (17)	N6—H6N	0.859 (16)
N3—C2	1.463 (4)	N7—C19	1.464 (4)
N4—C13	1.477 (4)	N8—C30	1.467 (3)
C1—C6	1.404 (4)	C18—C23	1.399 (4)
C1—C2	1.408 (4)	C18—C19	1.414 (4)
C2—C3	1.395 (4)	C19—C20	1.389 (4)
C3—C4	1.367 (4)	C20—C21	1.368 (4)
C3—H3	0.9300	C20—H20	0.9300
C4—C5	1.381 (4)	C21—C22	1.383 (4)
C4—H4	0.9300	C21—H21	0.9300
C5—C6	1.373 (4)	C22—C23	1.380 (4)
C5—H5	0.9300	C22—H22	0.9300
C6—H6	0.9300	C23—H23	0.9300
C7—C8	1.501 (4)	C24—C25	1.510 (4)
C8—C9	1.521 (3)	C25—C26	1.520 (3)
C8—H8A	0.9700	C25—H25A	0.9700
C8—H8B	0.9700	C25—H25B	0.9700
C9—C10	1.492 (4)	C26—C27	1.525 (4)
C9—H9A	0.9700	C26—H26A	0.9700
C9—H9B	0.9700	C26—H26B	0.9700
C10—C11	1.499 (4)	C27—C28	1.508 (3)
C10—H10A	0.9700	C27—H27A	0.9700
C10—H10B	0.9700	C27—H27B	0.9700
C12—C17	1.386 (4)	C29—C34	1.389 (3)
C12—C13	1.394 (4)	C29—C30	1.392 (3)
C13—C14	1.381 (4)	C30—C31	1.392 (4)
C14—C15	1.371 (4)	C31—C32	1.376 (4)
C14—H14	0.9300	C31—H31	0.9300
C15—C16	1.384 (4)	C32—C33	1.376 (4)
C15—H15	0.9300	C32—H32	0.9300
C16—C17	1.391 (4)	C33—C34	1.385 (4)
C16—H16	0.9300	C33—H33	0.9300
C17—H17	0.9300	C34—H34	0.9300
			
C7—N1—C1	128.9 (2)	C24—N5—C18	128.3 (2)
C7—N1—H1N	114 (2)	C24—N5—H5N	117 (2)
C1—N1—H1N	117 (2)	C18—N5—H5N	115 (2)
C11—N2—C12	124.6 (2)	C28—N6—C29	123.0 (2)
C11—N2—H2N	117.7 (19)	C28—N6—H6N	116.9 (18)
C12—N2—H2N	117.2 (19)	C29—N6—H6N	116.9 (18)
O3—N3—O4	120.3 (3)	O9—N7—O10	121.3 (3)
O3—N3—C2	118.9 (3)	O9—N7—C19	118.5 (3)
O4—N3—C2	120.7 (2)	O10—N7—C19	120.2 (2)
O5—N4—O6	124.0 (3)	O11—N8—O12	123.6 (2)
O5—N4—C13	118.4 (2)	O11—N8—C30	118.5 (2)
O6—N4—C13	117.7 (2)	O12—N8—C30	117.9 (2)
N1—C1—C6	121.9 (2)	N5—C18—C23	121.3 (2)
N1—C1—C2	121.7 (2)	N5—C18—C19	122.6 (2)
C6—C1—C2	116.4 (3)	C23—C18—C19	116.1 (3)
C3—C2—C1	121.4 (3)	C20—C19—C18	121.5 (3)
C3—C2—N3	116.0 (2)	C20—C19—N7	116.2 (3)
C1—C2—N3	122.6 (3)	C18—C19—N7	122.3 (3)
C4—C3—C2	120.5 (3)	C21—C20—C19	120.7 (3)
C4—C3—H3	119.7	C21—C20—H20	119.7
C2—C3—H3	119.7	C19—C20—H20	119.7
C3—C4—C5	119.0 (3)	C20—C21—C22	119.2 (3)
C3—C4—H4	120.5	C20—C21—H21	120.4
C5—C4—H4	120.5	C22—C21—H21	120.4
C6—C5—C4	121.3 (3)	C23—C22—C21	120.8 (3)
C6—C5—H5	119.4	C23—C22—H22	119.6
C4—C5—H5	119.4	C21—C22—H22	119.6
C5—C6—C1	121.4 (3)	C22—C23—C18	121.8 (3)
C5—C6—H6	119.3	C22—C23—H23	119.1
C1—C6—H6	119.3	C18—C23—H23	119.1
O1—C7—N1	123.5 (3)	O7—C24—N5	123.3 (3)
O1—C7—C8	123.6 (2)	O7—C24—C25	123.1 (2)
N1—C7—C8	112.9 (2)	N5—C24—C25	113.6 (2)
C7—C8—C9	114.8 (2)	C24—C25—C26	112.7 (2)
C7—C8—H8A	108.6	C24—C25—H25A	109.0
C9—C8—H8A	108.6	C26—C25—H25A	109.0
C7—C8—H8B	108.6	C24—C25—H25B	109.0
C9—C8—H8B	108.6	C26—C25—H25B	109.0
H8A—C8—H8B	107.6	H25A—C25—H25B	107.8
C10—C9—C8	111.1 (2)	C25—C26—C27	112.5 (2)
C10—C9—H9A	109.4	C25—C26—H26A	109.1
C8—C9—H9A	109.4	C27—C26—H26A	109.1
C10—C9—H9B	109.4	C25—C26—H26B	109.1
C8—C9—H9B	109.4	C27—C26—H26B	109.1
H9A—C9—H9B	108.0	H26A—C26—H26B	107.8
C9—C10—C11	116.5 (2)	C28—C27—C26	110.5 (2)
C9—C10—H10A	108.2	C28—C27—H27A	109.6
C11—C10—H10A	108.2	C26—C27—H27A	109.6
C9—C10—H10B	108.2	C28—C27—H27B	109.6
C11—C10—H10B	108.2	C26—C27—H27B	109.6
H10A—C10—H10B	107.3	H27A—C27—H27B	108.1
O2—C11—N2	121.9 (2)	O8—C28—N6	121.8 (2)
O2—C11—C10	124.2 (2)	O8—C28—C27	123.0 (2)
N2—C11—C10	113.8 (2)	N6—C28—C27	115.2 (2)
C17—C12—C13	116.7 (2)	C34—C29—C30	117.2 (2)
C17—C12—N2	119.8 (2)	C34—C29—N6	119.5 (2)
C13—C12—N2	123.5 (2)	C30—C29—N6	123.3 (2)
C14—C13—C12	122.5 (3)	C31—C30—C29	121.6 (3)
C14—C13—N4	116.1 (3)	C31—C30—N8	116.7 (2)
C12—C13—N4	121.2 (2)	C29—C30—N8	121.6 (2)
C15—C14—C13	119.9 (3)	C32—C31—C30	120.0 (3)
C15—C14—H14	120.1	C32—C31—H31	120.0
C13—C14—H14	120.1	C30—C31—H31	120.0
C14—C15—C16	119.1 (3)	C33—C32—C31	119.2 (3)
C14—C15—H15	120.5	C33—C32—H32	120.4
C16—C15—H15	120.5	C31—C32—H32	120.4
C15—C16—C17	120.7 (3)	C32—C33—C34	120.8 (3)
C15—C16—H16	119.6	C32—C33—H33	119.6
C17—C16—H16	119.6	C34—C33—H33	119.6
C12—C17—C16	121.1 (3)	C33—C34—C29	121.2 (3)
C12—C17—H17	119.4	C33—C34—H34	119.4
C16—C17—H17	119.4	C29—C34—H34	119.4
			
C7—N1—C1—C6	22.1 (5)	C24—N5—C18—C23	19.3 (4)
C7—N1—C1—C2	−159.5 (3)	C24—N5—C18—C19	−161.9 (3)
N1—C1—C2—C3	−178.7 (3)	N5—C18—C19—C20	−179.2 (3)
C6—C1—C2—C3	−0.2 (4)	C23—C18—C19—C20	−0.3 (4)
N1—C1—C2—N3	2.0 (4)	N5—C18—C19—N7	0.6 (4)
C6—C1—C2—N3	−179.4 (2)	C23—C18—C19—N7	179.5 (2)
O3—N3—C2—C3	1.1 (4)	O9—N7—C19—C20	−5.5 (4)
O4—N3—C2—C3	180.0 (3)	O10—N7—C19—C20	173.9 (3)
O3—N3—C2—C1	−179.5 (3)	O9—N7—C19—C18	174.7 (3)
O4—N3—C2—C1	−0.7 (4)	O10—N7—C19—C18	−5.9 (4)
C1—C2—C3—C4	−0.6 (4)	C18—C19—C20—C21	−0.3 (4)
N3—C2—C3—C4	178.7 (3)	N7—C19—C20—C21	179.9 (3)
C2—C3—C4—C5	0.7 (5)	C19—C20—C21—C22	−0.1 (5)
C3—C4—C5—C6	−0.1 (5)	C20—C21—C22—C23	1.0 (5)
C4—C5—C6—C1	−0.6 (5)	C21—C22—C23—C18	−1.6 (5)
N1—C1—C6—C5	179.3 (3)	N5—C18—C23—C22	−179.8 (3)
C2—C1—C6—C5	0.8 (4)	C19—C18—C23—C22	1.2 (4)
C1—N1—C7—O1	0.7 (5)	C18—N5—C24—O7	2.0 (5)
C1—N1—C7—C8	179.8 (3)	C18—N5—C24—C25	−178.7 (3)
O1—C7—C8—C9	−8.9 (4)	O7—C24—C25—C26	−13.5 (4)
N1—C7—C8—C9	172.0 (3)	N5—C24—C25—C26	167.2 (2)
C7—C8—C9—C10	−172.7 (3)	C24—C25—C26—C27	−176.7 (2)
C8—C9—C10—C11	−172.3 (3)	C25—C26—C27—C28	171.9 (2)
C12—N2—C11—O2	4.0 (4)	C29—N6—C28—O8	5.2 (4)
C12—N2—C11—C10	−172.6 (3)	C29—N6—C28—C27	−177.3 (2)
C9—C10—C11—O2	4.8 (5)	C26—C27—C28—O8	57.3 (4)
C9—C10—C11—N2	−178.7 (3)	C26—C27—C28—N6	−120.2 (3)
C11—N2—C12—C17	−131.7 (3)	C28—N6—C29—C34	127.7 (3)
C11—N2—C12—C13	48.6 (4)	C28—N6—C29—C30	−50.6 (4)
C17—C12—C13—C14	1.3 (4)	C34—C29—C30—C31	−1.9 (4)
N2—C12—C13—C14	−179.0 (2)	N6—C29—C30—C31	176.4 (2)
C17—C12—C13—N4	−175.0 (2)	C34—C29—C30—N8	174.8 (2)
N2—C12—C13—N4	4.7 (4)	N6—C29—C30—N8	−6.9 (4)
O5—N4—C13—C14	−131.3 (3)	O11—N8—C30—C31	134.3 (3)
O6—N4—C13—C14	46.7 (3)	O12—N8—C30—C31	−43.7 (3)
O5—N4—C13—C12	45.2 (4)	O11—N8—C30—C29	−42.5 (4)
O6—N4—C13—C12	−136.7 (3)	O12—N8—C30—C29	139.5 (3)
C12—C13—C14—C15	−0.9 (4)	C29—C30—C31—C32	1.2 (4)
N4—C13—C14—C15	175.5 (2)	N8—C30—C31—C32	−175.7 (3)
C13—C14—C15—C16	−0.3 (4)	C30—C31—C32—C33	0.3 (4)
C14—C15—C16—C17	1.2 (4)	C31—C32—C33—C34	−0.9 (5)
C13—C12—C17—C16	−0.4 (4)	C32—C33—C34—C29	0.1 (4)
N2—C12—C17—C16	179.9 (2)	C30—C29—C34—C33	1.3 (4)
C15—C16—C17—C12	−0.9 (4)	N6—C29—C34—C33	−177.1 (3)

### Hydrogen-bond geometry (Å, º)

**Table:**

*D*—H···*A*	*D*—H	H···*A*	*D*···*A*	*D*—H···*A*
N1—H1*N*···O4	0.86 (2)	2.01 (3)	2.639 (3)	130 (3)
N2—H2*N*···O8	0.86 (2)	2.17 (2)	3.002 (3)	162 (3)
C3—H3···O5^i^	0.93	2.49	3.337 (4)	151
C14—H14···O6^ii^	0.93	2.50	3.267 (3)	140
N5—H5*N*···O10	0.86 (2)	2.01 (3)	2.651 (3)	130 (3)
N6—H6*N*···O2^iii^	0.86 (2)	2.11 (2)	2.959 (3)	171 (3)
C27—H27*B*···O2^iii^	0.97	2.55	3.396 (3)	146
C31—H31···O12^iv^	0.93	2.49	3.238 (3)	138

Symmetry codes: (i) −*x*, −*y*+1, −*z*; (ii) −*x*, −*y*+2, −*z*+1; (iii) *x*, *y*−1, *z*; (iv) −*x*+1, −*y*+1, −*z*+1.
